# Background and emerging applications of acoustic metamaterials: a forum on classical waves

**DOI:** 10.1093/nsr/nwae366

**Published:** 2024-11-06

**Authors:** He Zhu

**Affiliations:** NSR, Beijing, China

## Abstract

Advancements in physical concepts and manufacturing technology such as 3D printing have generated an exciting field of research in recent years that is known as ‘metamaterials’. When applied to electromagnetic waves, novel applications have emerged in sensing or cloaking devices. In contrast, acoustic metamaterials may provide solutions to acoustic problems as well as a conceptual and developmental platform for condensed-matter physics. National Science Review invited Prof. Hong Chen of Tongji University to organize a forum to discuss this unique field of acoustic metamaterials in physical research.

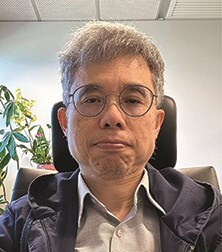

**Che Ting Chan** (陈子亭)

Professor, Department of Physics, Hongkong University of Science and Technology

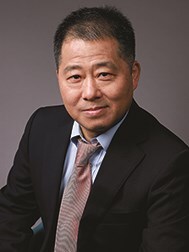

**Yan-Feng Chen** (陈延峰)

Professor, College of Engineering and Applied Sciences, Nanjing University

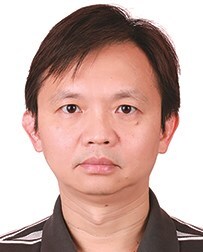

**Zhengyou Liu** (刘正猷)

Professor, School of Physical & Electronic Sciences, Wuhan University

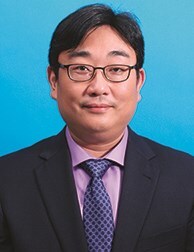

**Jie Zhu** (祝捷)

Professor, School of Physical Science and Engineering, Tongji University

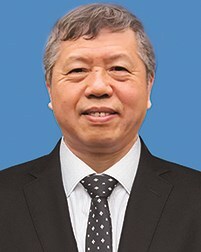

**Hong Chen** (陈鸿) (Chair)

Professor, School of Physical Science and Engineering, Tongji University

## Teaser

Acoustic metamaterials provide new dimensions over traditional materials to modulate sound wave. As a result, we observe new phenomenon and achieve new functions. This is a Forum article, please use the corresponding template.

**Chen H:** Metamaterials are now receiving considerable attention from researchers, as this field is growing rapidly at the intersection of many scientific disciplines. Young scholars whom I meet at conferences on metamaterials have expressed to me their enthusiasm at learning about new theories and applications, particularly about acoustic metamaterials. So, today, we have invited several renowned experts in this field, including Prof. Che Ting Chan of Hongkong University of Science and Technology, Prof. Yan-Feng Chen of Nanjing University, Prof. Zhengyou Liu of Wuhan University and Prof. Jie Zhu of Tongji University.

Let's start our discussion with an example. When freeways run through cities, we see boards of patterned holes along both sides as sound barriers that protect city environments from freeway noise. Acoustic metamaterials, similar to sound barriers,

Acoustic metamaterials provide new dimensions over traditional materials to modulate sound waves. As a result, we observe new phenomena and achieve new functions.

—Hong Chen

are artificial materials with microstructures. What is their relation to traditional acoustic materials? Furthermore, what is the difference between acoustic metamaterials and electromagnetic metamaterials? Prof. Liu, would you like to start with these questions?

They (acoustic metamaterials) may possess properties that do not exist in nature, such as negative mass density, negative elastic modulus and negative reflective index.

—Zhengyou Liu

**Figure 1.**
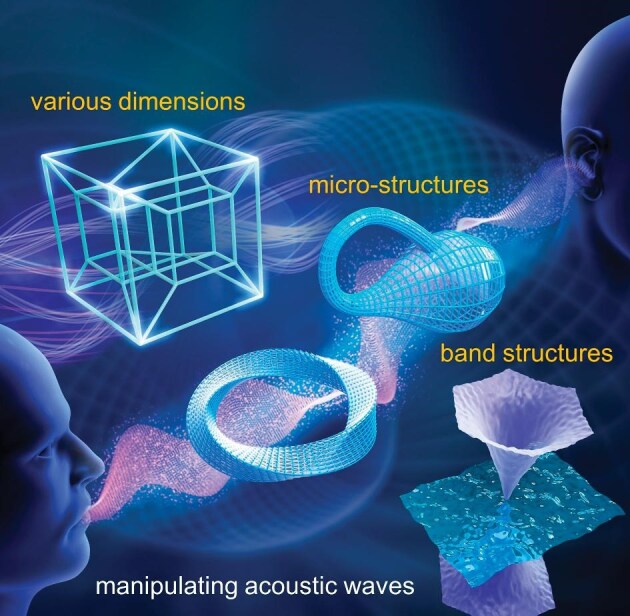
Manipulation of acoustic waves with artificial microstructures as well as their band structures (*Courtesy of Prof. Yan-Feng Chen*).

**Liu ZY:** Traditional acoustic materials originate from natural materials with fixed parameters, so their acoustic properties are difficult to change or modulate. Acoustic metamaterials are artificially designed and manufactured with microstructures so that their acoustic parameters may exceed values of natural materials and they may possess properties that do not exist in nature, such as negative mass density, negative elastic modulus and negative reflective index. These properties may realize exotic phenomena such as perfect imaging and acoustic cloaking. In comparison, electromagnetic metamaterials are designed and manufactured to expand materials’ electromagnetic parameters, such as dielectric constant and magnetic permeability. Electromagnetic metamaterials aim to modulate electromagnetic waves or light to exceed natural materials in related applications.

**Chen H:** Next, let's discuss the challenges facing traditional acoustic materials. Prof. Chen, would you like to describe them and how they may be overcome by new methods of modulation enabled by acoustic metamaterials?

**Figure 2.**
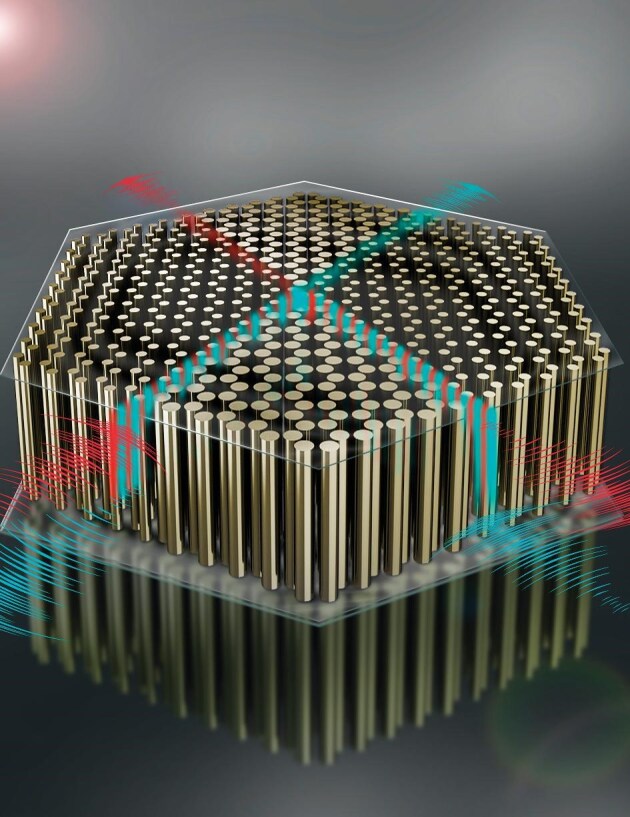
Schematic of 2D quantum spin Hall effect for airborne sound (*Courtesy of Prof. Yan-Feng Chen*).

**Chen YF:** Traditional acoustic materials such as foam and rubber have wide applications in construction and civil engineering as sound absorbers. However, traditional materials have problems that are difficult to overcome. For example, sound absorption at low frequencies has been challenging for traditional materials, as they require extended thickness and space to match long wavelengths. So, using these materials for noise reduction and vibration isolation at low frequencies remains ineffective, particularly in airplanes and in boats, where space is limited. Another challenge is sound reduction of extreme intensities, at amplitudes of ∼150 dB. Jet pilots are greatly affected by this, as their helmets or ear mufflers need to reduce the intensity to below 120 dB, otherwise it is severely detrimental to their health.

Metamaterials with artificially defined microstructures may be designed for one device to contain multiple modes so it may resonate over an expanded frequency range.

—Yan-Feng Chen

An example in the high-frequency range is devices for surface acoustic waves that are commonly used in smart phones. These traditional devices rely on resonating units usually with fixed center frequencies, so their operating bandwidth is limited. Metamaterials with artificially defined microstructures may be designed for one device to contain multiple modes so it may resonate over an expanded frequency range. Our domestic technology in this area currently lags that in foreign imports so we hope new design concepts and mechanisms in metamaterials may provide an opportunity to catch up. We have made significant progress in recent years with modern methods in information science and manufacturing. Specifically, machine learning improves the algorithms to design and optimize these devices and 3D printing now allows us to realize complex microstructures.

**Chen H:** We have mentioned that metamaterials enable exotic methods to modulate materials’ properties. One powerful dimension of modulation is through band structures in the energy–momentum plots. This aspect of metamaterials research originates from condensed-matter physics in which electron band structures play a central role in determining the properties of a particular crystal. One hot topic in condensed-matter physics is topological materials. Now, the concept of band topology of electrons has been adapted and found applications in classical waves. So my question to Prof. Che Ting Chan is: What is different about topological acoustics compared with topological electronics and topological photonics?

**Figure 3.**
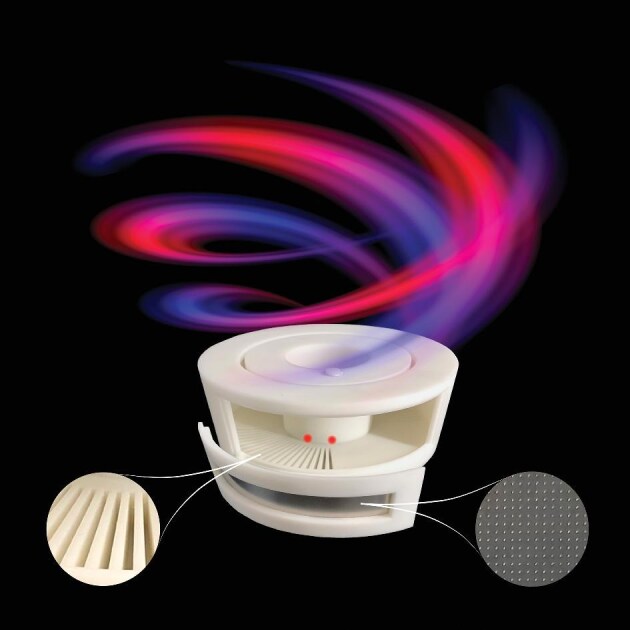
Chirality-switchable vortex sound beam generated by parity-time symmetric acoustic metamaterial crystal ring cavity (*Courtesy of Prof. Jie Zhu*).

**Chan CT:** When we describe a periodic physical system with a unit cell, we find energy bands with corresponding eigenstates. We can define topological characteristics for each eigenstate. In the case of energy bands of electrons, the topology of band structures may exhibit special properties, such as winding numbers and Chern numbers. These topological features have analogs in acoustics due to the common underlying mathematics. However, there are several differences between electronic and acoustic systems. Firstly, sound waves are bosonic when quantized, as opposed to the fermionic nature of electrons. This means the corresponding symmetry consequences can be different. In electronic systems, we typically focus only on the Fermi surface, which defines the level up to which electrons can occupy. States away from the Fermi surface are less relevant. In contrast, all frequency bands are meaningful in an acoustic system. Additionally, electronic systems are many-body systems that are frequently approximated as one-body systems in mean-field-type band-structure descriptions, while acoustic systems are classical and do not require many-body corrections. Another key difference is that photonic waves are intrinsic vector waves. While 1D and 2D forms of photonic waves can be treated with a scalar approximation, this cannot be used for 3D electromagnetic waves. In comparison, acoustic waves are longitudinal, making acoustics a more manageable platform on which to study topological phenomena. Finally, electronic systems must be defined according to the actual elements in the periodic table, while acoustic systems can be more arbitrarily defined, including examples such as ‘half an atom in the unit cell or a rotated atom in the unit cell’. The topological properties in electronic and acoustic systems share a common mathematical foundation, but the physical differences between the two lead to distinct challenges and advantages in their respective fields of study.

**Chen H:** Given the special properties of topological acoustic materials, what advantages can be realized in applications? Prof. Liu, can you answer this question?

**Figure 4.**
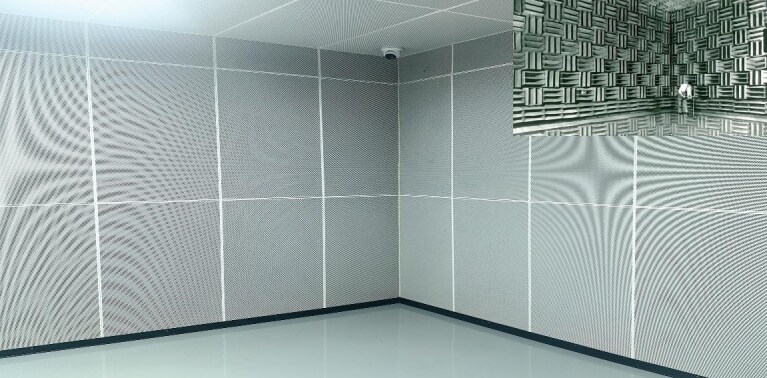
Semi-anechoic chamber built with acoustic metamaterials by China Acoustics Valley in 2022 and earliest semi-anechoic chamber (inset) built by Bell's Lab in 1947 (*Courtesy of Prof. Yan-Feng Chen*).

**Liu ZY:** As topological electronic materials are based on natural materials, their topological properties often exist in limited ranges. Specific examples include small and fixed bandgaps of topological insulators and difficulties in obtaining ideal topological semimetals. As acoustic metamaterials are artificially designed, they can be customized to present all imaginable topological properties. This aspect cannot be matched by electronic or even photonic materials. First, as sound waves are scalar longitudinal waves with simple eigen modes, it is more likely to design acoustic metamaterials to satisfy complex requirements. Second, acoustic wavelengths are in a macroscopic range, so modern manufacturing technologies such as 3D printing, computerized machining and optical etching can all be employed to realize innovative designs. Third, due to the bosonic nature of phonons, topological acoustic metamaterials may function in multiple frequency bands: one material can be designed to function as a topological insulator at one frequency and a topological semimetal at another frequency. Lastly, acoustic metamaterials can be designed to demonstrate unnatural properties. In an electronic system, generally, nearest-neighbor interactions are stronger than second-nearest-neighbor interactions, and second-nearest-neighbor interactions are stronger than third-nearest-neighbor interactions, and so on. But, in an acoustic system, we can design a structure in which long-range interactions are stronger than short-range interactions. In terms of breakthrough applications, the best possibility may emerge as a device with topological surface acoustic waves.

**Chen H:** Application scenarios of acoustic metamaterials start with sound waves in air and include more complex cases such as ultrasound and water waves. When designing and manufacturing metamaterials and metasurfaces in these special cases, what special principles should be followed? Prof. Zhu, would you like to discuss this problem?

**Zhu J:** The development of acoustic metamaterials in the past 20 years has been mostly focused on materials interacting with sound waves in air. This is certainly natural, as air is the environment of our daily lives and audible sound is the medium of our communications and information exchange. As other experts have pointed out, as sound waves in air are longitudinal scalar waves with simple modes of propagation, early successes in the design and manufacturing of these materials quickly emerged. Alongside applications such as materials for noise reduction in air, materials to work with water waves and solid vibrations have also been designed. Applications in water are particularly significant, as electromagnetic waves face strong damping when propagating in water. Consequently, sound waves are now the primary candidate of exchange for information and energy in water. As the technologies to design metamaterials extend from audible sound waves in air to ultrasound in water, basic principles will continue to apply but we will face some unique challenges. First, the speed of sound in water is much higher than sound in air. Second, the impedances of sound waves in air and in water also differ greatly. When designing metamaterials in air, the impedances of air and the solid material are so far separated that the surfaces of the solid can be treated as hard boundaries. In water, on the other hand, the coupling between solids and water as the medium must be considered, as water and solids have similar impedances. In addition, the choices of durable solid materials in water are limited due to the possibility of erosion by the water. Metamaterials working with ultrasound need to contain structures that are comparable to much shorter wavelengths, at the micrometer range and below. This is beyond the capability of common manufacturing technologies, such as 3D printing and machining. Fortunately, recent advances in fabrication technologies in the micrometer or nanometer range have enabled and improved metamaterials working in the ultrasound range. In conclusion, applications of metamaterials will significantly grow from sound waves in air to ultrasound in water and solids. The obstacles met in these new areas will be overcome one by one and soon we will see the emergence of new metamaterials.

**Figure 5.**
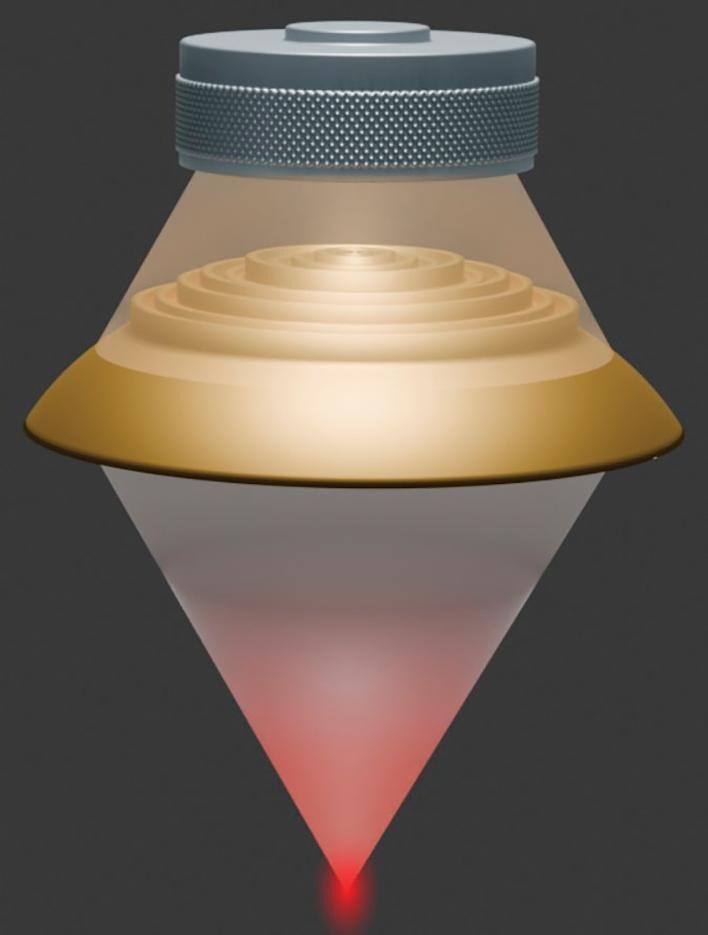
An inversely designed metalens, optimized using a genetic algorithm, can significantly enhance ultrasound transmission through skull-like rigid materials while providing focusing function (*Courtesy of Prof. Jie Zhu*).

**Chen H:** Several experts have emphasized that modern manufacturing technologies facilitated the development of acoustic metamaterials. On a conceptual level or in terms of basic design principles, what are the characteristics of acoustic metamaterials?

**Chen YF:** We mentioned several branches of acoustic research in air, in water and in solids. Traditional air-based acoustic research focuses on voice processing and noise reduction; water-based research focuses on sonar and impedance matching; solid-based research focuses on bulk or surface acoustic waves and vibration control. The wavelengths of these problems cover a range as large as 10 orders of magnitude, so they are treated as separate problems. As we are facing the same collection of problems when using metamaterials, we treat them by using a systematic framework with concepts such as periodic structures, energy band topology and effective medium theory. This general and unified approach to a wide range of problems may explain why this field is now growing to be so vibrant and exciting, as a new solution to one problem may likely inspire progress in the entire field.

**Chen H:** Next, let's discuss a special case of metamaterial called metafluid. Prof. Liu, what is metafluid and what is unique about this material?

**Liu ZY:** Unlike metamaterials designed for electromagnetic waves, acoustic metamaterials can be further classified according to their material background as fluid or solid. In fluids such as air and water, acoustic waves are scalar longitudinal waves, whereas waves propagating in solids are vector waves. Metamaterials with a fluid background are called metafluids. Acoustic properties of fluids in nature are characterized by positive mass density and bulk modulus. If we introduce artificial structures into fluids, then we may create properties that do not exist in natural fluids. If the fluid-based artificial structures allow monopolar resonances, the bulk modulus of the fluid may become negative. If the structures allow dipolar resonances, the mass density of the fluid may become negative. If we further create quadrupolar resonances into metafluid, it may accommodate an effective shear-like modulus to behave like a solid and allow transverse waves to propagate. New applications based on this type of metafluid may emerge in acoustic cloaking, acoustic waveguide and acoustic focusing with flat surfaces.

**Chen H:** Artificial intelligence (AI) and machine learning (ML) are currently the hottest topics in scientific research. In designing microstructures for metamaterials, will AI eventually become the primary tool?

Now, we are often witnessing AI with a backward logic using existing solutions as training data and generating new solutions without an apparent ‘understanding’ of the problem.

—Che Ting Chan

**Chan CT:** I think that scenario happening is just a matter of time. When we humans want to realize a material's function, we tend to rely on a forward logic, using physical and mathematical principles that we understand and our experience. Now, we are often witnessing AI with a backward logic using existing solutions as training data and generating new solutions without an apparent ‘understanding’ of the problem. In the next phase of AI design, we may specify desired functions and constraints, and AI will generate structures that will likely exceed humans’ intuition. Let's use topology optimization as an example. Decades ago, when civil engineers aimed to design airplanes to be as light as possible, even before the advent of AI, optimization using genetic algorithms exceeded human designs. So, if we view metamaterial design as a problem of microstructure optimization, better algorithms combined with better computation capacity such as more GPUs will certainly defeat human brains.

**Chen H:** What can AI contribute to real-world applications of acoustic metamaterials?

**Zhu J:** I agree with Prof. Chan that new technologies such as AI and ML will make increasing contributions to the development of acoustic metamaterials. In the past decades, we have mostly relied on a forward design workflow that starts with the design of a unit cell, then build a numerical model and analyse energy bands. By evaluating the characteristics of the energy bands, we achieve the desired modulation of sound waves. The advantage of the forward design method is that we build on an understandable mechanism and realize a function. However, this approach may be less effective with a complex sound modulation function or a combination of multiple functions if we cannot propose a proper starting point. An inverse design with AI and big data may be crucial in solving problems beyond humans’ comprehension, especially when the design process needs to include the coupling of multiphysics fields, such as sound, heat and light. As AI technologies, including generative AI, rapidly improve, the inverse method will become more convenient and powerful in difficult areas such as multidimensional optimizations.

When we first introduce AI or ML to metamaterials design, we likely face a shortage of training data, as these methods are known to be ‘data-hungry’, much like the famous Alpha-fold problem.

—Jie Zhu

We should certainly mention the challenges that we currently face when we transition to AI technologies. When we first introduce AI or ML to metamaterials design, we likely face a shortage of training data, as these methods are known to be ‘data-hungry’, much like the famous Alpha-fold problem. In addition, many other professions similar to ours are also exploring AI solutions so the availability of computation resources is unfortunately limited for everybody. A third challenge is that generative AI as a problem solver is much like a ‘black box’. We specify desired functions as an input and get a microstructure design as an output, but AI tools do not provide any explanation or summary of the design process. To us scientists, understanding the mechanism behind a certain design is extremely beneficial so AI's inability to help with that, at the moment, is considered a major shortfall. Another minor issue is that AI-generated microstructure designs may present difficulties for manufacturing capability, but I think improving technologies will alleviate that.

**Chen H:** Can we introduce some commercialization potentials of acoustic metamaterials?

**Zhu J:** Medical applications of ultrasound such as High Intensity Focused Ultrasound (HIFU) present the focusing of control-related problems that may be alternatively addressed with metasurfaces or metalenses. Current HIFU solutions rely on multiple piezoelectric elements, of which amplitudes, phases and locations can be modulated to achieve focusing. Instead, we may replace arrays of small piezo elements with one large piezo element combined with a metasurface or metalens. The microstructures on a metalens can be designed to modulate amplitudes and phases spatially, and achieve a function of focusing. A series of such metalenses can be designed and manufactured to provide focusing at multiple depths.

**Chen YF:** Noise reduction in advanced transportation is an area in which we expect to see successes of acoustic metamaterials. As high-speed trains in China are becoming the fastest in the world,

the requirement for noise reduction has also reached an unprecedented level. We are collaborating with the Railway Administration to meet this challenge. Another example is the COMAC-C919 commercial jet produced domestically. For commercial jets, passenger comfort is becoming an important feature of competition. We are also collaborating with the manufacturer of C919 to build this plane into an example of domestic design and engineering.

**Figure 6.**
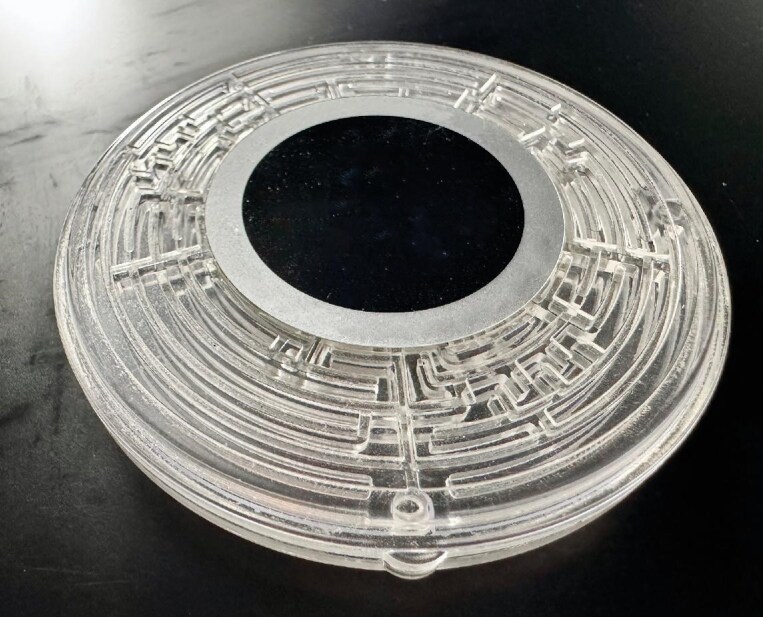
Maze-like metamaterial absorber used in a compact speaker design (*Courtesy of Prof. Jie Zhu*).

**Zhu J:** I'd like to mention another example. Acoustic Metamaterials Group from Hong Kong collaborated with a world-renowned company, KEF, to bring acoustic metamaterials to consumer electronics. They designed internal sound absorbers for audio speakers by using metamaterial technology, so these speakers can be much thinner and lighter than traditional types. Their first model on the market that uses metamaterials technology is called LS50-Meta.

**Chen H:** Our last topic is on the future directions in acoustic metamaterials. What new concepts and principles will create the next generation of metamaterials technology?

**Chen YF:** First, non-linearity may become a future direction in acoustics, as non-linear optics is now an important area of innovation. Other emerging topics include parity-time symmetry, non-Hermitian systems and the coupling between phonons and electrons or photons. In addition, the quantum mechanical properties of phonons in a bosonic system can be further explored. At high frequencies, as wavelengths decrease, particularly in cryogenic systems, phonons may function as a method of control in next-generation quantum computational devices.

**Chan CT:** The acoustic systems that we have studied so far have been passive in nature. As we begin to explore active acoustic systems, we may uncover new and intriguing phenomena. From a quantum mechanical perspective, one potential future direction could be the ability to modulate the phonon spectrum and the interaction between phonons and electrons. If we can control the phonon spectrum through structural modulation, we may be able to influence electron–phonon coupling, which could have consequences for effects such as superconductivity.

**Chen H:** In summary, acoustic metamaterials provide new dimensions over traditional materials to modulate sound waves. As a result, we observe new phenomena and achieve new functions. Now, as we take full advantage of AI technologies, we see great potential for advanced technologies and breakthrough applications. This concludes today's exciting discussion and thank you all for your contribution.

